# Computational study of nitro-benzylidene phenazine as dengue virus-2 NS2B-NS3 protease inhibitor

**DOI:** 10.3389/fmolb.2022.875424

**Published:** 2022-11-17

**Authors:** Nurul Hanim Salin, Maywan Hariono, Nur Sarah Dyana Khalili, Iffah Izzati Zakaria, Fadi G. Saqallah, Mohamad Nurul Azmi Mohamad Taib, Ezatul Ezleen Kamarulzaman, Habibah A. Wahab, Muhammad Hidhir Khawory

**Affiliations:** ^1^ Malaysian Institute of Pharmaceuticals and Nutraceuticals, National Institutes of Biotechnology Malaysia, Gelugor, Pulau Pinang, Malaysia; ^2^ School of Pharmaceutical Sciences, Universiti Sains Malaysia, Gelugor, Pulau Pinang, Malaysia; ^3^ School of Chemical Sciences, Universiti Sains Malaysia, Gelugor, Pulau Pinang, Malaysia; ^4^ Malaysia Genome and Vaccine Institute, National Institutes of Biotechnology Malaysia, Kajang, Selangor, Malaysia

**Keywords:** pharmacophore model, molecular docking analysis, antiviral drug discovery, DENV2, dengue cases

## Abstract

According to the World Health Organisation (WHO), as of week 23 of 2022, there were more than 1,311 cases of dengue in Malaysia, with 13 deaths reported. Furthermore, there was an increase of 65.7% during the same period in 2021. Despite the increase in cumulative dengue incidence, there is no effective antiviral drug available for dengue treatment. This work aimed to evaluate several nitro-benzylidene phenazine compounds, especially those that contain 4-hydroxy-3,5-*bis*((2-(4-nitrophenyl)hydrazinylidene)-methyl)benzoate through pharmacophore queries selection method as potential dengue virus 2 (DENV2) NS2B-NS3 protease inhibitors. Herein, molecular docking was employed to correlate the energies of selected hits’ free binding and their binding affinities. Pan assay interference compounds (PAINS) filter was also adopted to identify and assess the drug-likeness, toxicity, mutagenicity potentials, and pharmacokinetic profiles to select hit compounds that can be considered as lead DENV2 NS2B-NS3 protease inhibitors. Molecular dynamics assessment of two nitro-benzylidene phenazine derivatives bearing dinitro and hydroxy groups at the benzylidene ring showed their stability at the main binding pocket of DENV2 protease, where their MM-PBSA binding energies were between -22.53 and -17.01 kcal/mol. This work reports those two nitro-benzylidene phenazine derivatives as hits with 52–55% efficiency as antiviral candidates. Therefore, further optimisation is required to minimise the lead compounds’ toxicity and mutagenicity.

## 1 Introduction

Dengue infection, also known as DENV infection, is a serious and global viral health concern because of its morbidity and mortality rates. These rates are because there are no proper treatments for severe dengue infection available at present (Kan et al., 2020). Dengue virus can be transmitted mainly through *Aedes aegypti* and *Aedes albopictus* mosquitoes. Dengue virus is a *Flavivirus* that belongs to the Flaviviridae family with a positive-strand RNA (Wahab et al., 2007).

The dengue genome comprises three structural proteins and seven non-structural (NS) proteins. The three structural proteins include the nucleocapsids, also known as core (C) protein, a membrane-associated (M) protein, and an envelope (E) glycoprotein. In contrast, the seven non-structural (NS) proteins include NS1, NS2A, NS2B, NS3, NS4A, NS4B, and NS5 (Bell et al., 1985). There are four antigenically distinct but closely related serotypes of dengue virus, mainly DENV1, DENV2, DENV3, and DENV4, that exhibit almost 65%–70% of the sequence homology. To date, dengue fever has been reported to be triggered by these four different serotypes. The fifth variant, DENV5, was first isolated in October 2013, and it follows the sylvatic cycle, unlike the other four serotypes which follow the human cycle. The possible cause of the emergence of the new serotype could be genetic recombination, natural selection, and genetic bottlenecks (Mustafa et al., 2015).

NS3 is a trypsin-like serine protease with a catalytic triad made up of HIS51, ASP75, and SER135, and its activity is enhanced by NS2B as the cofactor. This cofactor contributes to the NS3 activity through its hydrophilic region that is responsible for holding and promoting the activation of NS3. In contrast, the hydrophobic region takes part in the membrane association during the cleavage process (Yap et al., 2019). The non-structural protein NS2B-NS3 serine protease plays an important role in the replication of *Flavivirus*, which is required for the synthesis of polyprotein precursors. Therefore, this protease is considered to be an essential target for the development of anti-DENV drugs.

**FIGURE 1:**
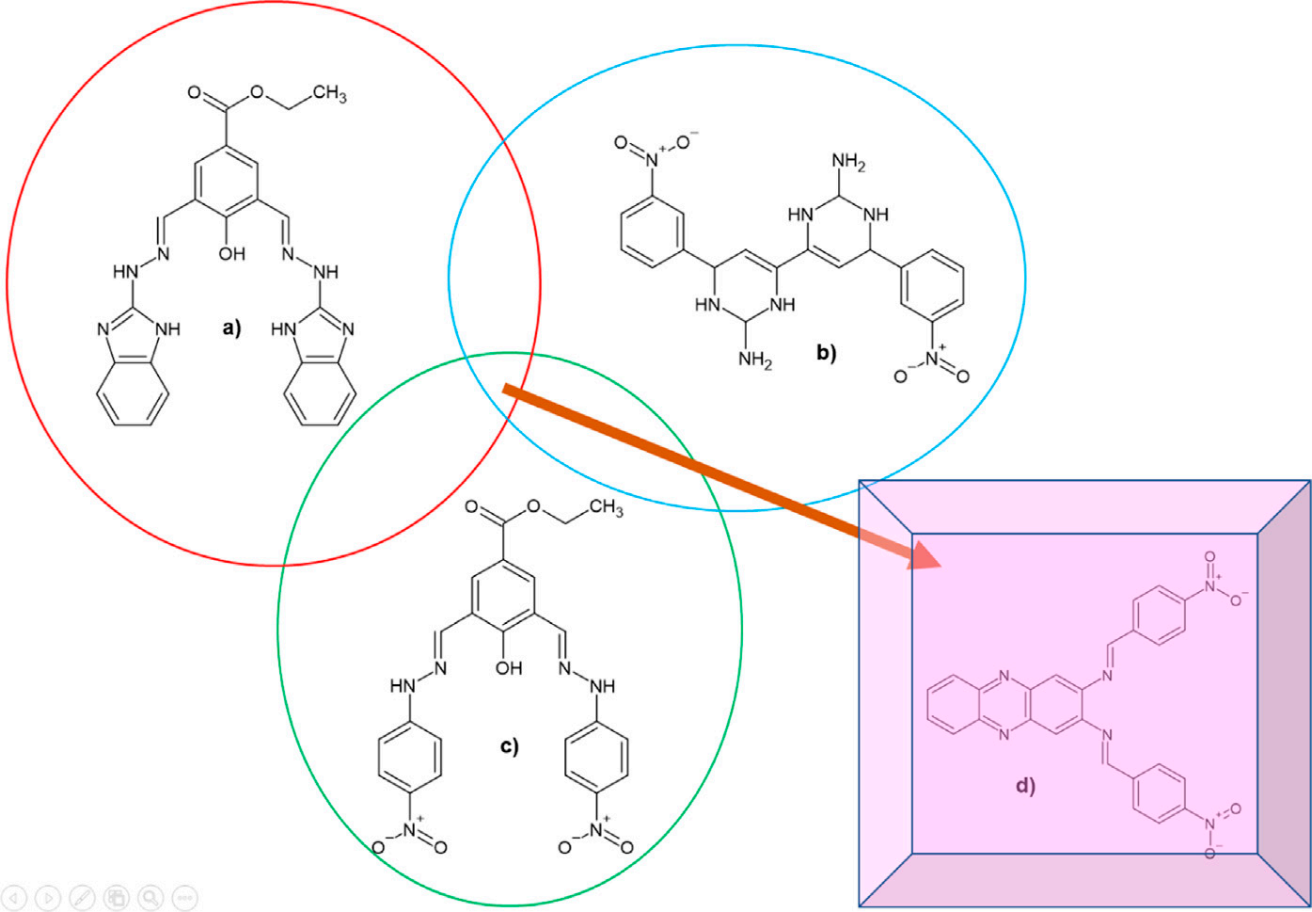
2D chemical structures of **(A)** the lead compound (**1**; IC_50_ = 13.1 µM), **(B) 1855** (IC_50_ = 29.41 µM), **(C) 22a** (IC_50_ = 14.58 µM), and **(D)** the suggested compound to be designed by extracting the common functional group in the previous known active compounds against DENV2 NS2B-NS3 protease.

Previous studies reported quinoline, benzimidazole, and thioguanine, as DENV2 NS2B-NS3 protease inhibitors (Deng et al., 2012; Hariono et al., 2019). It was suggested that an *N*-heterocycle in the compound’s structure offers a significant scaffold that might contribute to the inhibitory activities of DENV2 NS2B-NS3 protease. Deng and co-workers identified one potential hit from the ethyl 4-hydroxy-3,5-*bis*((2-(4-nitrophenyl)hydrazinylidene)-methyl)benzoate (**22a**) scaffold (Figure 1), which exhibited an IC_50_ of 14.58 µM (Deng et al., 2012). In addition, the inhibitory activity of the *N*-heterocyclic dimer of compounds attached with 3-nitrophenyl demonstrated protease inhibitory activity with an IC_50_ of 29.41 µM. Based on these findings, selected key structures containing nitrophenyl, hydrazinylidene, and the *N*-heterocycle were subjected to complex-based pharmacophore computational modeling. Important pharmacophoric features towards determining structural requirements for dengue protease inhibitor development and its feasibility to be synthesised on a laboratory scale were elucidated. The pharmacophore-based virtual screening obtained a nitro-benzylidene phenazine derivatives library, which was then selected to screen for lead inhibitors toward DENV2 NS2B-NS3 protease. These derivatives were then synthesised employing the Schiff base reaction, which is considered a cost-effective reaction feasible for upscaling.

Computer-aided drug design (CADD) and its application in virtual screening can be used to screen for a hit compound and further optimise it into a lead (Hariono et al., 2021). In this work, a pharmacophore model was generated based on the docked structure of **22a** into the DENV2 NS2B-NS3 protease binding site. In contrast with the conventional ligand-based pharmacophore approach, which cannot provide detailed structural information during lead optimisation, the structure-based pharmacophore provides an alternative approach to address the ligand-based pharmacophore limitation. Despite the recently reported DENV protease-inhibitor complexes at the allosteric site (Yildiz et al., 2013; Yao et al., 2019), the unavailability of a crystal structure of the NS2B-NS3 protease in complex with an inhibitor at its catalytic pocket hinders the adaptation of this approach. Therefore, to obtain appropriate binding of **22a** (IC_50_ = 14.58 µM) in the active site of DENV2 NS2B-NS3 protease, molecular docking was conducted and followed by structure-based pharmacophore generation using Ligand Scout (Inte:Ligand GmbH, Vienna, Austria). Using the heuristic approach and template-based numeric analysis, the ligand is perceived and interpreted according to a probable molecular topology along with the hybridisation states and bond types from (often ambiguous) geometric information (Gaurav and Gautam, 2014). Subsequently, the generated pharmacophore was validated against active and decoy compounds, as detailed in the methodology. Upon retrospective validation, the pharmacophore model was used as queries to screen hits from the nitro-benzylidene phenazine database. These hits were then studied in terms of their binding mode, true positive potential inhibitor, drug-likeness, potential toxicity and mutagenicity, and pharmacokinetic profiles.

Recently, two novel inhibitors (DC-RA016 and DC-RA052) were successfully identified through structure-based drug design against SARS-CoV-2 S-RBD/ACE2, which then were experimentally verified (Xiong et al., 2021). In addition, MaleLiu et al., 2021 described developing a high-throughput screening model as a critical technology to screen for S protein-ACE2 blockers, focusing on virtual screening to offer concepts for rapid discovery (MaleLiu et al., 2021). Moreover, successful predictions of stability and binding affinities of the 11 (re)designed nanobody scaffolds (Nbs) based on the structures of the five Nbs in complex with protein receptor-binding domain (RBD), suggest that the appropriate computational approach can help in the design of efficient SARS-CoV-2 infection inhibitors (Yang et al., 2021). Besides, a computational study employing conformation-based virtual screening uncovered SARS-CoV-2 Mpro inhibitors based on molecular dynamics and the binding free energy analysis (Yang et al., 2021).

## 2 Methods

### 2.1 Hardware and software

A laptop equipped with AMD^®^ Ryzen 3 2200U, VGA Radeon^®^ Vega 3, 4 GB RAM, and 1TB HDD, and a workstation with Intel^®^ Xeon^®^ E5-2620 v3, NVIDIA GeForce GTX TITAN X, 64 GB RAM, and 1 TB HDD. Software employed include Marvin Sketch (www.chemaxon.com), AutoDock 4.2.6, and AutoDockTools 1.5.6 (www.autodock.scripps.edu), LigandScout 4.4.7 (www.inteligand.com), BIOVIA Discovery Studio 2020 (www.accelrys.com), AMBER MD 2018 (www.ambermd.org), and UCSF Chimera 1.15 (www.cgl.ucsf.edu/chimera/).

### 2.2 Molecular docking

DENV2 NS2B-NS3 protease homology model by Wichapong and co-workers (2010) (JMR_977_sm_SupplMat1.pdb) (Wichapong et al., 2010) was prepared by assigning Kollman charges using AutoDockTools 1.5.6. The grid box was 60 × 60×60 in size with 0.375 Å spacing and centred at *x* = 21.517, *y* = 43.428, *z* = -1.743. Compound **22a (**4-hydroxy-3,5-*bis*((2-(4-nitrophenyl)hydrazinylidene)-methyl)benzoate), which demonstrated an IC_50_ of 14.58 µM experimentally, was utilised as the ligand. The ligand was protonated using BIOVIA Discovery Studio 2020 and assigned with Gasteiger Charges using AutoDockTools 1.5.6. Docking for 250 Lamarckian Genetic Searching Algorithm runs was carried out using AutoDock 4.2.6 with the same parameters reported in our previous study (Hariono et al., 2019). The protein-ligand interactions were visualised using BIOVIA Discovery Studio 2020.

### 2.3 Structure-based pharmacophore design

The docked conformation of **22a** towards the active site of DENV2 NS2B-NS3 protease was uploaded to LigandScout 4.4.7, and the pharmacophores were generated using a structure-based pharmacophore tool. The external validation was carried out utilising two sets of ligands retrieved from literature (Deng et al., 2012) (see Supplementary Tables S1, S2). All these ligands were *in vitro* tested against DENV2 NS2B-NS3 protease *via* FRET-based assay under the same biological conditions. They classified the IC_50_’s between 7.46 and 41.24 µM as active, whereas ligands with IC_50_ of more than 50 µM were defined as a decoy. The ligands (Table 1) were screened into pharmacophore using the screening tool with the following parameters: scoring functions = pharmacophore-fit; max numbers of omitted features = 5; compounds timeout = 0 min; screening mode: match all query features; retrieval mode = stop after first matching conformation; execution mode = multi-threaded. Upon validation, the pharmacophore models were used as the filter to identify the testing ligands (the nitro-benzylidene phenazine derivatives) and whether they have pharmacophoric features that are close/similar to the reference ligand (**22a**).

**TABLE 1:** The thirteen nitro-benzylidene phenazine derivatives used in the hits screening.

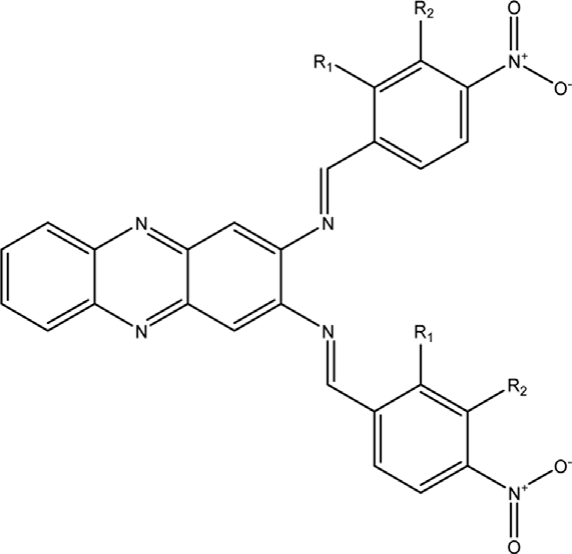		
Ligand	R_1_	R_2_
1	H	H
2	C_2_H_5_	H
3	Cl	H
4	H	NO_2_
5	OH	H
6	OCH_3_	H
7	OC_2_H_5_	H
8	OC_3_H_7_	H
9	OC_4_H_9_	H
10	COCH_3_	H
11	COC_2_H_5_	H
12	COC_3_H_7_	H
13	COC_4_H_9_	H

### 2.4 PAINS filter

The screened compounds using structure-based pharmacophores were checked for the requirement to pass the pan assay interference compounds (PAINS) test (http://cbligand.org/PAINS/) before proceeding with further testing and analyses (Baell & Holloway, 2010).

### 2.5 ADMETox prediction

The Lipinski’s Rule, mutagenicity, toxicity and pharmacokinetic prediction were carried out using the pkCSM online tool (https://structure.bioc.cam.ac.uk/pkcsm) (Pires et al., 2015).

#### 2.5.1 Lipinski’s Rule of Five

The Lipinski’s Rules profile was individually assessed using the pkCSM online tool server. Parameters which were predicted include the molecular weight (MW), partition coefficient (logP), the number of hydrogen bond donors (HBD), the number of hydrogen bond acceptors (HBA), the number of rotatable bonds, and the surface area.

#### 2.5.2 Mutagenicity and toxicity studies

Using the same protocol employed in Lipinski’s Rule of Five, the mutagenic potencies of the ligands were predicted *via* the AMES test by the pkCSM online tool. In addition, other parameters such as the maximum tolerated dose (human) (hMTD), ether-a-go-go related gene (human) (hERG) I inhibitor, hERG II inhibitor, oral rat acute toxicity (logLD_50_), oral rat chronic toxicity (logLOAEL), hepatotoxicity, skin sensitisation, *T. pyriformis* (*TP*) toxicity, and minnow toxicity represented the toxicity properties of the ligands.

#### 2.5.3 Pharmacokinetics study

Using the same approach used in Lipinski’s Rule of five, the ADME (absorption, distribution, metabolism, and excretion) profiles of the ligands were predicted *via* the pkCSM online tool. The absorption is influenced by water solubility, Caco2 permeability, skin permeability, P-glycoprotein substrate, P-glycoprotein I inhibitor, and P-glycoprotein II inhibitor, instead of human gastrointestinal absorption. The distribution was represented by volume distribution-steady state (VDss) (human), fraction unbound (human), blood-brain barrier (BBB) permeability, and central nervous system (CNS) permeability. The metabolism was represented by the cytochrome P450 2D6 (CYP2D6) substrate, the CYP3A4 substrate, CYP1A2 inhibitor, CYP2C19 inhibitor, CYP2C9 inhibitor, CYP2D6 inhibitor, and CYP3A4 inhibitor. Lastly, the excretion was represented by total clearance and renal organic cation transporter 2 (OCT2) substrate.

### 2.6 Molecular dynamics evaluation

The stability of compounds **4** and **5** at the catalytic binding pocket of NS2B-NS3 protease was evaluated using molecular dynamics simulations using AMBER MD 2018 (Case et al., 2018). The binding conformations of both ligands (i.e., **4** and **5**) which possess the lowest ΔG_bind_ from the molecular docking study were used as starting coordinates using the same method described in our earlier work with minor modifications (Hariono et al., 2019). Briefly, the two systems were prepared using the LEaP program assigning the ff14SB force field for the protein and General AMBER Force Field (GAFF) to the ligands. The systems were solvated in the TIP3P water model (cubic box; 10.0 Å^3^) after neutralising them with one sodium cation (Na^+^). To relax the water molecules and eliminate any possible steric clashes, the steepest descent minimisation was applied (5,000 steps), followed by a conjugated gradient (5,000 steps). The systems were heated through a multistep process, gradually raising the temperature of the systems from 0° to 310 K using the canonical ensemble (NVT), followed by equilibration for 3 ns using the isothermal-isobaric ensemble (NPT). Production was executed for a total of 50 ns as a multistep process (5 × 10 ns) with a Langevin-thermostat collision frequency γ at 1 ps^−1^. Trajectories of the simulations were analysed using CPPTRAJ for their root mean square deviation (RMSD), root mean square fluctuation (RMSF), a radius of gyration (RadGyr), and H-bond interactions (Roe and Cheatham, 2013) and visualised with the help of UCSF Chimera 1.15 (Goddard et al., 2005). Lastly, 500 frames from the last 5 ns of the simulations were analysed for their bonded and non-bonded energy components using the MM-PBSA python module as part of the AMBER 18 package (Miller et al., 2012).

## 3 Results

**FIGURE 2:**
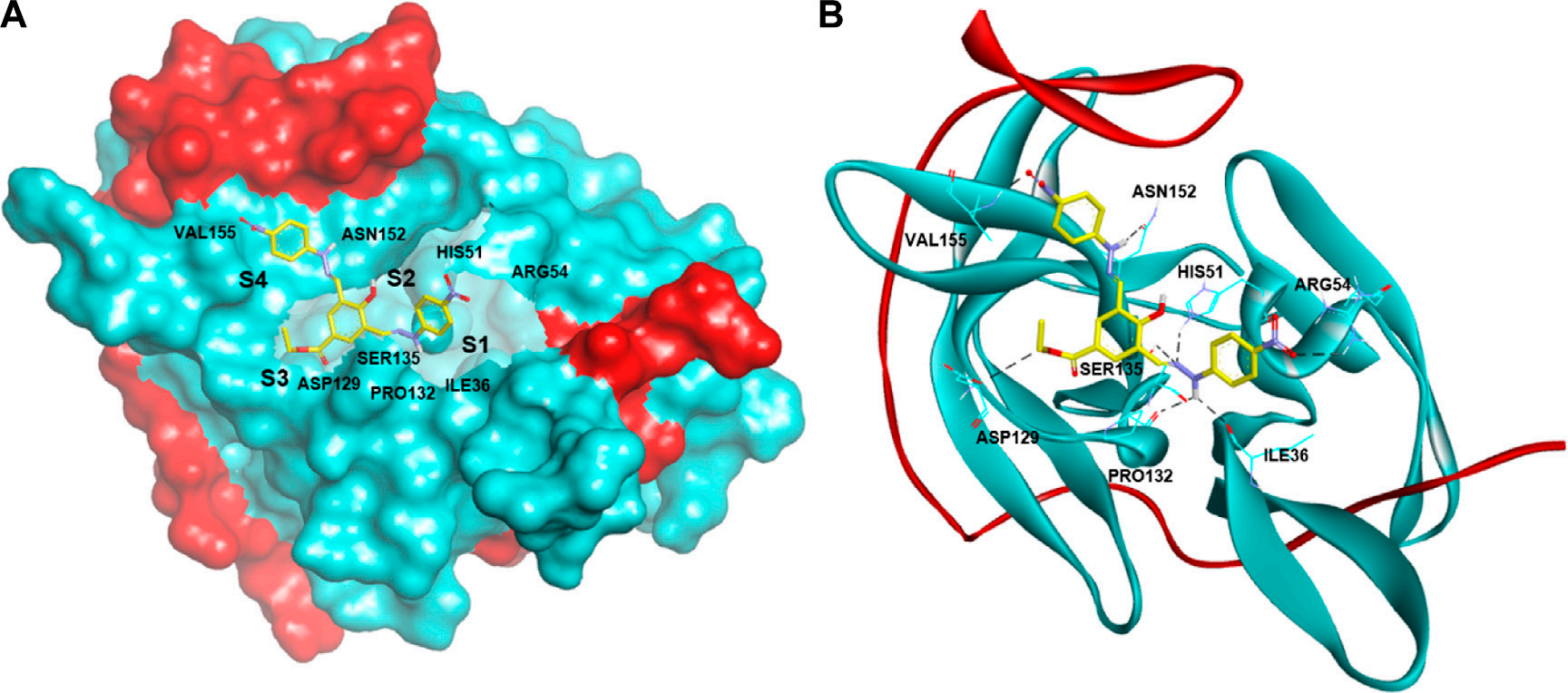
Docking of active compound **22a** having quinoline scaffold against DENV2 NS2B-NS3 protease with ΔG_bind_ of -9.36 kcal/mol in **(A)** surface and **(B)** ribbon protein models. The red and cyan areas represent the NS2B and NS3 protease chains, respectively. The H-bond interactions are shown as black dashed lines.

The docking of the reference compound **22a** showed a ΔG_bind_ of -9.36 kcal/mol (Deng et al., 2012), which is slightly lower than compound **1855** (-8.90 kcal/mol) (Hariono et al., 2019). Considering the docking error of ±2.0 kcal/mol, these two compounds have a comparable binding affinity towards DENV2 NS2B-NS3 protease. The binding mode of **22a** depicted in Figure 2, shows H-bond interactions with the S1 pocket (HIS51, SER135, and ASP129), S2 pocket (ASN152), and S4 pocket (VAL155 and ILE36). Other H-bond interactions with non-specific pockets also occur with PRO132 and ARG54. In the S4 pocket, the NO_2_ group interacts with VAL155, whereas hydrazine NH interacts with ASN152 of the S2 pocket. The other hydrazine NH is exposed to the S1 pocket by interacting with SER135. In addition, the hydrazine N = interacts with HIS51, whereas the ethoxy ester O interacts with ASP129 at the S1 pocket. Although it is not explicitly exposed to the sub-pocket, another NO_2_ group and the hydrazine NH interact with ARG54 and PRO132, respectively. These binding modes describe the initial pharmacophore feature of **22a** that potentially inhibits the DENV2 NS2B-NS3 protease. Figure 2 illustrates the docking profile of **22a** in the binding pocket of DENV2 NS2B-NS3 protease.

**FIGURE 3:**
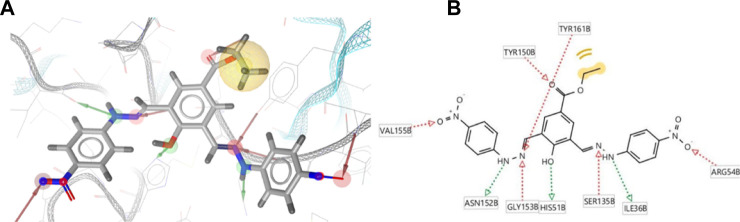
Structure-based pharmacophore generation of **22a** employing 5 hydrogen bond acceptors (HBA; red), 3 hydrogen bond donors (HBD; green), and 1 hydrophobic (yellow) feature in **(A)** 3D and **(B)** 2D visualisations.

The pharmacophore of **22a** was successfully generated using a structure-based pharmacophore method. As initially determined by the molecular docking, the common features of the pharmacophores are identified as follows: five hydrogen bond acceptors (HBA) at NO_2_ (N), hydrazine (N = ), and carbonyl’s O with the number of features of 2, 2, and 1, respectively; three hydrogen bond donors (HBD) at hydrazine (NH), OH, with the number of features of 2 and 1, respectively; and one hydrophobic at ethyl group. These common pharmacophoric features strongly agree with the binding mode demonstrated by molecular docking. The 3D structure of **22a** pharmacophore queries is presented in Figure 3.

The pharmacophore model of **22a** forms a heptagonal shape with inter-distances from feature to feature as follows: HBA-H (4.19 Å), H-HBA (8.54 Å), HBA-HBA (7.17 Å), HBA-HBD (1.32 Å), HBA-HBD (4.07 Å), HBD-HBA (7.89 Å), HBA-HBD (6.24 Å), and lastly HBD-HBA (1.32 Å). These distances represent the relative positions of the functional groups that are essential for the binding affinity with the DENV2 NS2B-NS3 protease. Before the pharmacophore model is carried out in the screening for hits, a retrospective validation must be done to confirm the capability of the model to predict whether the compounds are true positive or false positive (Hariono et al., 2021).

**FIGURE 4:**
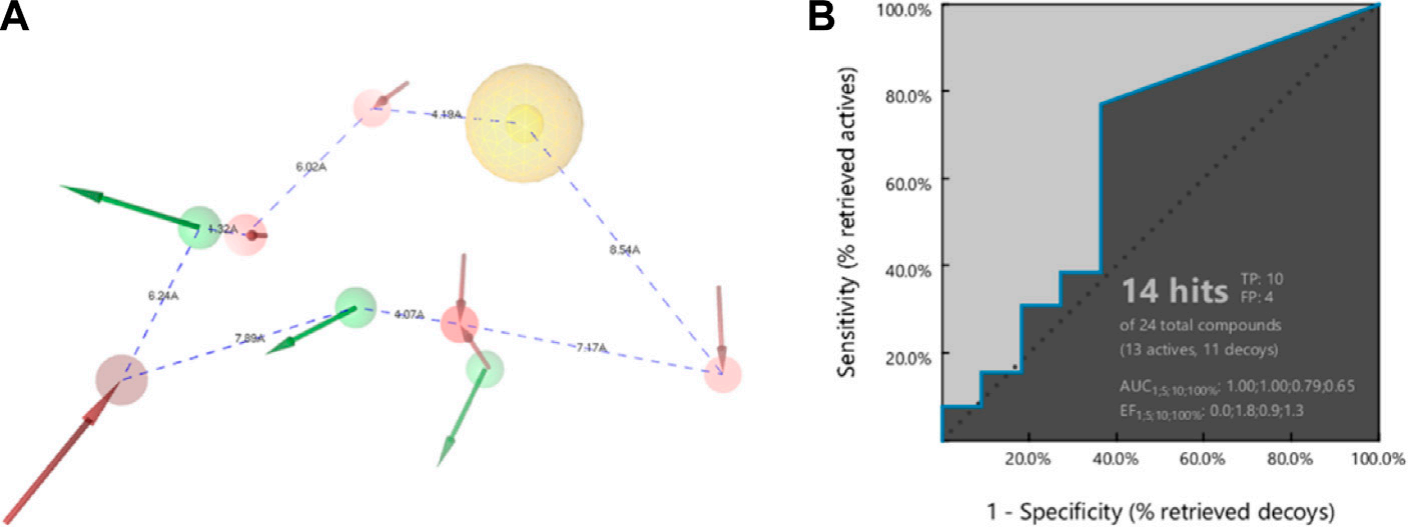
The pharmacophore **(A)** shape of **22a,** and **(B)** the ROC of external validation showing AUC 79% (threshold 10) and 65% (threshold 100). The Enrichment Factors are 0.9 and 1.3 times greater than expected for active compounds at thresholds of 10 and 100%, respectively.

A series of compounds that had been tested for their inhibitory activity against DENV2 NS2B-NS3 protease with the IC_50_s ranging from 7.46 to 41.24 µM was categorised as an active set. In contrast, a series of compounds showing IC_50_ > 50 µM against the corresponding enzyme was categorised as a decoy set. The result shows that the pharmacophore model can predict 10 out of 13 true positive compounds and 4 out of 11 false positive compounds. This means that the pharmacophore model has a strong predictive capability against unknown compounds regarding its activity against DENV2 NS2B-NS3 protease. The receiver operating characteristic (ROC) curve (Figure 4), showed AUC values of 1.00, 1.00, 0.79, and 0.65 at 1, 5, 10, and 100%, respectively. Since these AUC values are close to 100%, they are considered good values. In addition, the Enrichment Factors (EF) reflect that in the thresholds 1, 5, 10 and 100%, the active compound was found at 0, 1.8, 0.9 and 1.3, respectively, times greater than expected. Thus, based on the AUC and EF values, the pharmacophore model has a strong hypothesis to predict hits that act as DENV2 NS2B-NS3 protease inhibitors.

**FIGURE 5:**
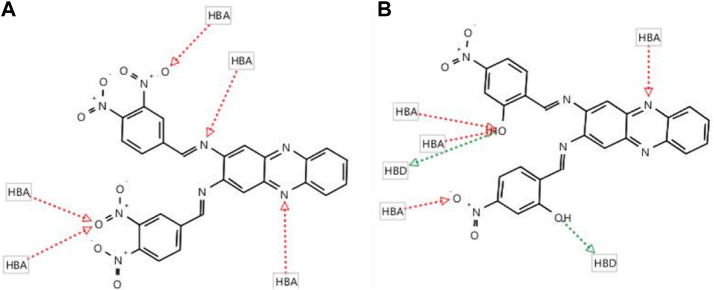
The screening results of nitro-benzylidene phenazine derivatives show two fit compounds **(A) 4** (55.84) and **(B) 5** (55.11) in the pharmacophore queries of **22a** in the binding pocket of DENV-2 NS2B-NS3 protease.

Subsequently, two hits, compounds **4** (fit score = 55.84) and compound **5** (fit score = 55.11) were identified (Figure 5). Although these two compounds could only fit in at least 50% relative position of the pharmacophore model, their potency can still be optimised as leads for DENV2 NS2B-NS3 protease inhibitors. The common pharmacophoric features of compound **4** which fit the model are 5 HBAs at NO_2_ (O), hydrazine N = , and heterocyclic N. Compound **5** has slightly different common features as it can fit with 3 HBAs at OH (O), NO_2_ (O), and heterocyclic N. In addition, compound **5** possesses 2 HBDs at OH (H).

**FIGURE 6:**
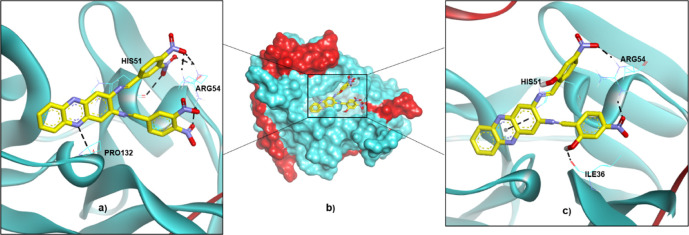
The binding conformations of compounds **4** and **5** in the binding pocket of DENV2 NS2B-NS3 protease are represented by **(A) 4** in a ribbon protein model, **(B) 4** and **5** in a surface protein model, and **(C) 5** in a ribbon protein model.

The two hits were then investigated for their ΔG_bind_ and binding modes *via* molecular docking. As presented in Table 2, the ΔG_bind_ of compound **4** (-9.48 kcal/mol) is almost similar to the value of the reference compound (**22a**; -9.36 kcal/mol). Thus, it can be assumed that these two compounds might also have DENV2 NS2B-NS3 protease inhibitory activities. However, H-bond interactions are only observed with ARG54 and PRO132, and the estimated K_i_ value is 0.11 µM. The predicted K_i_ is much lower than the experimental IC_50_ of 14.58µM, indicating that compound **4** might be more active than **22a**. On the other hand, compound **5** shows higher ΔG_bind_ (-7.72 kcal/mol) than those of **4** and **22a**. However, this energy was contributed by diverse H-bond interactions with ILE36, HIS51 and ARG54, resulting in the estimated K_i_ equal to 2.21 µM, which demonstrates high potency as shown by compound **4**. Figure 6 illustrates the binding conformations of compounds **4** and **5** in the binding pocket of DENV-2 NS2B-NS3 protease.

**TABLE 2:** The docking results of compounds **4** and **5** in the binding pocket of DENV2 NS2B-NS3 protease.

Ligand	ΔG_bind_ (kcal/mol)	H-bond interacting residue	Estimated K_i_ (µM)
4	−9.48	ARG54, PRO132	0.11
5	−7.72	ILE36, HIS51, ARG54	2.21

**FIGURE 7:**
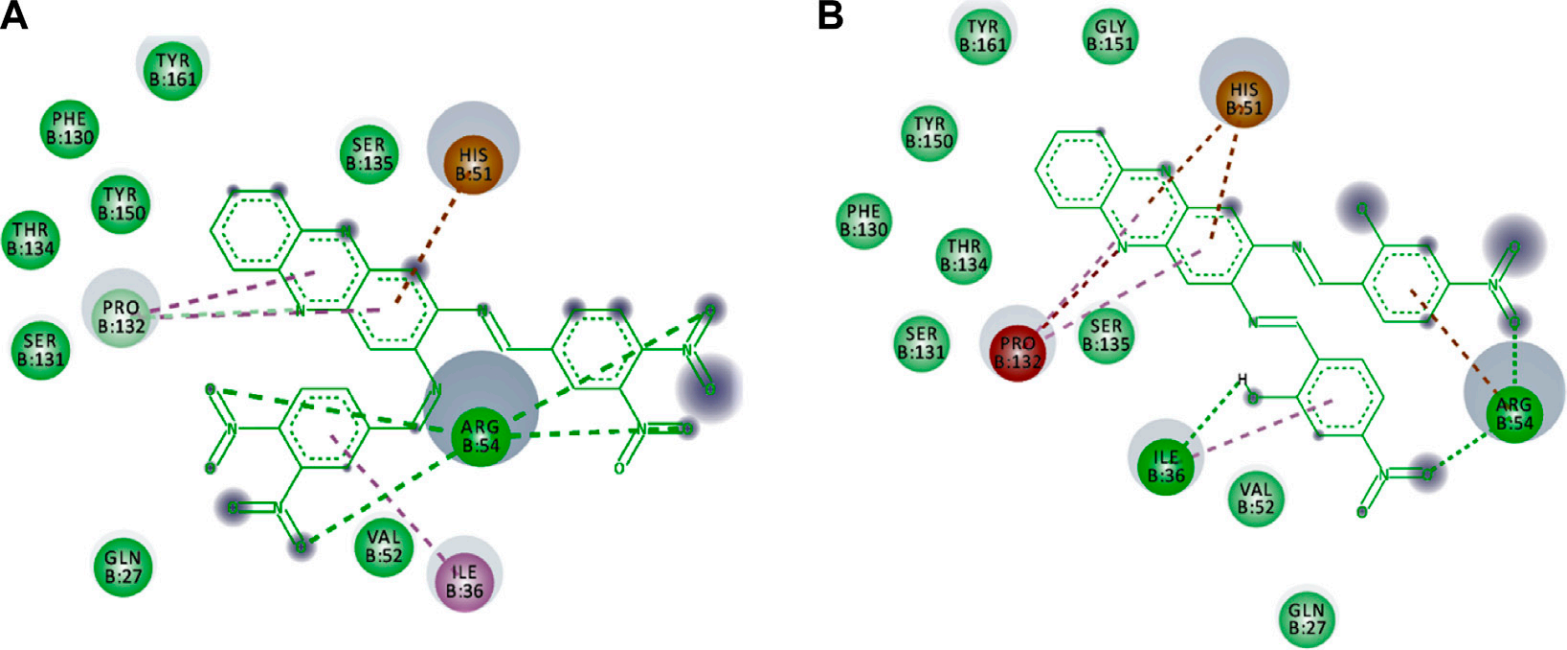
The non-bonding type environment of **(A)** compounds 4 and **(B)** 5 which might support their docked conformations. The colours of deep green, green, pale green, brown, magenta, pink, and grey represent vdW, H-bond, C-H bond, pi-cation, pi-pi-T shaped, pi-alkyl, and solvent exposures and interactions, respectively

The non-bonding interactions such as van der Waals (vdW), unfavourable acceptor-acceptor (UAA), pi-cation and pi-alkyl also support the binding of the compounds in the active site that lead to the stable conformation. As summarised in Table 3, vdW interactions are major non-bonding interactions in both selected hits. Other residues such as ARG54, PRO132, HIS51 and ILE36 are also involved in the non-bonding type, in addition to major contributions provided through H-bond interactions. Figure 7 illustrates the non-bonding type environment of compounds **4** and **5,** which might support their docked conformations.

**TABLE 3:** The non-bonding type of interactions of compound **4** and compound **5** in the binding pocket of DENV2 NS2B-NS3 protease.

Ligand	vdW	UAA	Pi-cation	Pi-alkyl
4	VAL52, GLN27, SER131, THR134, TYR150, PHE130, TYR161, SER135	na	HIS51	ILE36, PRO132
5	GLN27, VAL52, SER135, SER131, THR134, PHE130, TYR150, TYR161, GLY151	PRO132	ARG54, HIS51	PRO132

Na = not applied

The selected hits also passed the PAINS filter; thus, they might not give a false positive result due to the interference such as aggregating compounds, covalent bonding, and chelate formation when tested *in vitro* (Baell & Holloway, 2010). The hits are said to possess a drug-like structure when they meet the requirement as postulated by Lipinski’s Rule of Five, in which drugs should have a maximum of 500 g/mol MW, 5 for LogP, 5 for HBD, and 10 for HBA (Lipinski, 2004). Other drug-like structure rules also require that the drug have a maximum of 10 rotatable bonds and 140 in the surface area. Therefore, according to Table 4, both hits are slightly over in the MW, LogP and surface area.

**TABLE 4:** The drug-like structure evaluation results of the hits.

Ligand	MW	LogP	HBD	HBA	Rotatable bonds	Surface area
4	566.446	5.917	8	12	0	232.481
5	508.45	5.5118	6	10	2	212.763

The red colour indicates that the ligand does not meet the criteria of the corresponding parameter.

The mutagenic and toxic potency of compounds were predicted by their AMES toxicity (No), hMTD (>0.477), hERG 1 and hERG 2 inhibitor (No), ORAT and LOAEL (>2000 mg/kg, hepatotoxicity (No), skin sensitisation (No), TP toxicity (>0.5) and Minnow toxicity (>-0.3) (Huerta E, 2007; Barlow et al., 2009; Sorell, 2016; McCormick, 2017; Gadaleta et al., 2019; Hariyono et al., 2021). From the results presented in Table 5, both hits are likely to be potentially toxic and mutagenic, raising an alarm that the further process would need structural optimisation. On one hand, the optimisation of the structure should maintain the activity toward DENV2 NS2B-NS3 protease, but on the other hand, the toxicity and mutagenicity potential should be reduced accordingly.

**TABLE 5:** The mutagenic potency and toxicity evaluation result of the hits.

Ligand	AMES toxicity	hMTD	hERG I inhibitor	hERG II inhibitor	ORAT (logLD_50_)	ORCT (log LOAEL)	Hepatotoxicity	Skin sensitisation	*TP* toxicity	Minnow toxicity
4	Yes	0.468	No	Yes	2.659	−1.768	Yes	No	0.285	−9.215
5	Yes	0.307	No	Yes	2.531	2.583	Yes	No	0.285	−0.499

The red colour indicates that the ligand does not meet the criteria of the corresponding parameter.

Good absorption is one of the most important pharmacokinetic profiles that the hits should meet. Both hits showed similar absorption profiles (Table 6), in which they meet the requirement for water solubility (>-4), human intestinal absorption (100%), and skin permeability (<-2), with no action as the Pgp substrate. It should be noted, however, that **5** does not pass the criteria set for Pgp substrate as this compound potentially acts as a substrate for this protein. In addition, both compounds fail Caco2 permeability (<0.9) and PgpI and PgP II inhibitors criteria (Lin & Yamazaki, 2003; de Angelis and Turco, 2011; Hariyono et al., 2021).

**TABLE 6:** The absorption profile evaluation results of the hits.

Ligand	Water solubility	Caco2 permeability	Intestinal absorption (human)	Skin permeability	P-glycoprotein substrate	P-glycoprotein I inhibitor	P-glycoprotein II inhibitor
4	−3.417	−0.68	100	−2.735	No	Yes	Yes
5	−3.507	−0.257	100	−2.375	Yes	Yes	Yes

The red colour indicates that the ligand does not meet the criteria of the corresponding parameter

The second pharmacokinetic profile should be passed by the distribution of the hits. Table 7 shows that **4** does not meet two criteria, i.e., VDss (human), since the criteria should be > -0.15, and CNS permeability (<-3). Moreover, **5** fails in three criteria as it does not meet the requirement of BBB permeability, which should be < -1 (Ballabh et al., 2004). Interestingly, both hits passed the fraction unbound in humans, in which the values are above 0.15. Overall, **4** shows a better distribution compared to **5**.

**TABLE 7:** The distribution profile evaluation results of the hits.

Ligand	VDss (human)	Fraction unbound (fu) (human)	BBB permeability	CNS permeability
4	−1.421	0.345	−2.405	−2.156
5	−1.733	0.274	−0.975	−2.067

The red colour indicates that the ligand does not meet the criteria of the corresponding parameter.

Metabolism is another important pharmacokinetic profile, which most likely changes the active drug to its inactive metabolite (Kinirons and O’Mahony, 2004). Table 8 shows that compound **4** demonstrates a better metabolism profile than compound **5**. However, both are neither CYP2D6 substrates nor inhibitors. Furthermore, **4** does not act as CYP1A2 and CYP3A4 inhibitors. In contrast, **5** acts as the substrate of CYP3A4, CYP1A2 inhibitor, CYP2C19 inhibitor, CYP2C9 inhibitor and CYP3A4 inhibitor. This indicates that both hits can induce or inhibit a certain CYP, which might result in either the under-dose or over-dose of another drug metabolised by that certain CYP.

**TABLE 8:** The metabolism profile evaluation results of the hits.

Ligand	CYP2D6 substrate	CYP3A4 substrate	CYP1A2 inhibitor	CYP2C19 inhibitor	CYP2C9 inhibitor	CYP2D6 inhibitor	CYP3A4 inhibitor
4	No	Yes	No	Yes	Yes	No	No
5	No	Yes	Yes	Yes	Yes	No	Yes

The red colour indicates that the ligand does not meet the criteria of the corresponding parameter.

Excretion, in which the metabolite is mostly eliminated from the body *via* urine, is also an important predictive pharmacokinetic profile (Rosenbaum, 2016). Compound **4** is predicted to be excreted from the body at a faster rate than **5** due to its highest total clearance. In contrast, **5** might undergo slower removal from the body due to its lowest total clearance. Despite this, both hits have low total clearance (logCl < 0.763), and it is generally desirable to develop a drug for oral administration without a high dosage regimen (Toutain et al., 2004). Interestingly, none of the hits is predicted to act as renal OCT2 substrate, which might lead to undesirable side effects (Burt et al., 2016). Table 9 presents the total clearance of all hits that reflects their speed to be eliminated from the body system.

**TABLE 9:** The excretion profile evaluation results of the hits.

Ligand	Total clearance (mL/mnt/kg)	OCT2 substrate
4	0.438	No
5	0.008	No

**FIGURE 8:**
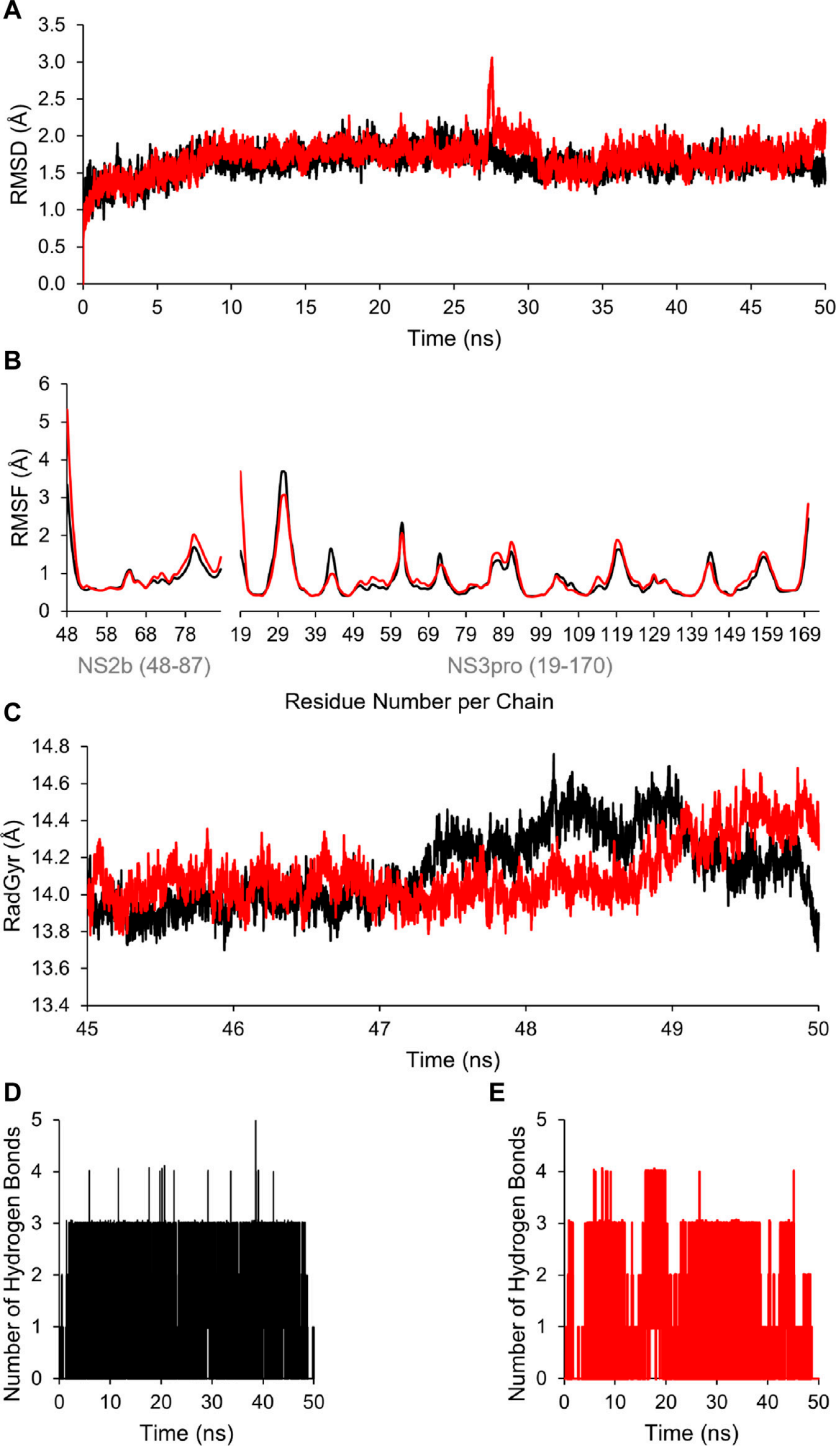
**(A)** Time evolution for the RMSD of NS2B/NS3 protease in complexes with compounds **4** (black) and **5** (red). **(B)** RMSF values of NS2B/NS3 protease residues when in complex with compounds **4** (black) and **5** (red). **(C)** Radius of gyration (RadGyr) of NS2B/NS3 protease in complex with compounds **4** (black) and **5** (red). **(D,E)** Number of H-bonds formed between the amino acid residues at the catalytic binding site of NS2B/NS3 protease when in complex with compounds **4** (black) and **5** (red), respectively.

**FIGURE 9:**
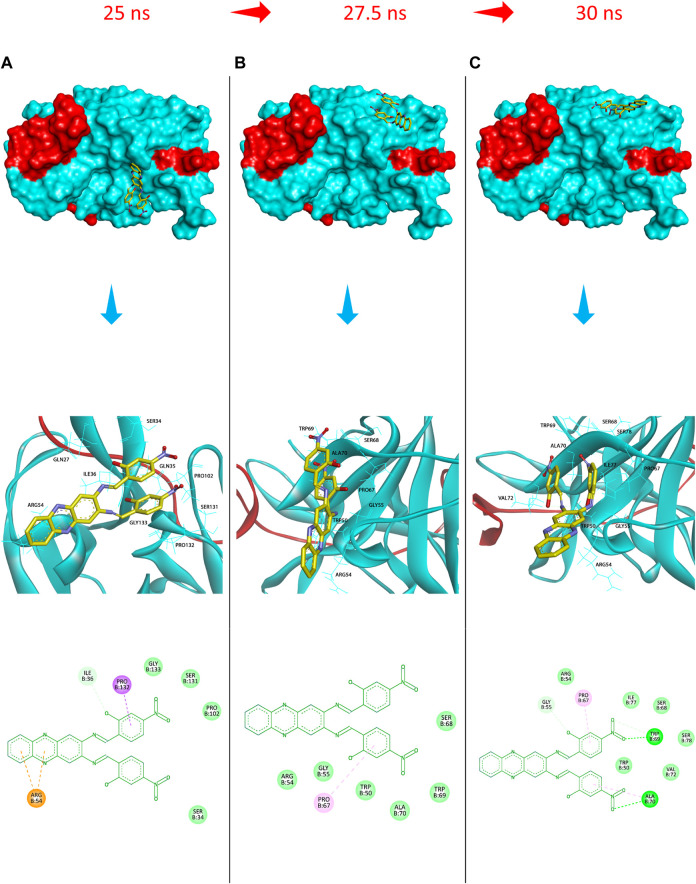
Protein surface, 3D, and 2D representations of the binding conformations of compound **5** (stick representation with yellow carbons) at the catalytic binding site of DENV2 NS2B-NS3 protease (ribbon representation; cyan for the NS3pro chain and red for the NS2B chain) at **(A)** 25 ns towards the S1 sub-pocket, **(B)** 27.5 ns towards the S2 sub-pocket, and **(C)** 30 ns as it maintains its binding towards the S2 sub-pocket. Frames were extracted using UCSF Chimera 1.15 and represented using BIOVIA Discovery Studio.

**FIGURE 10:**
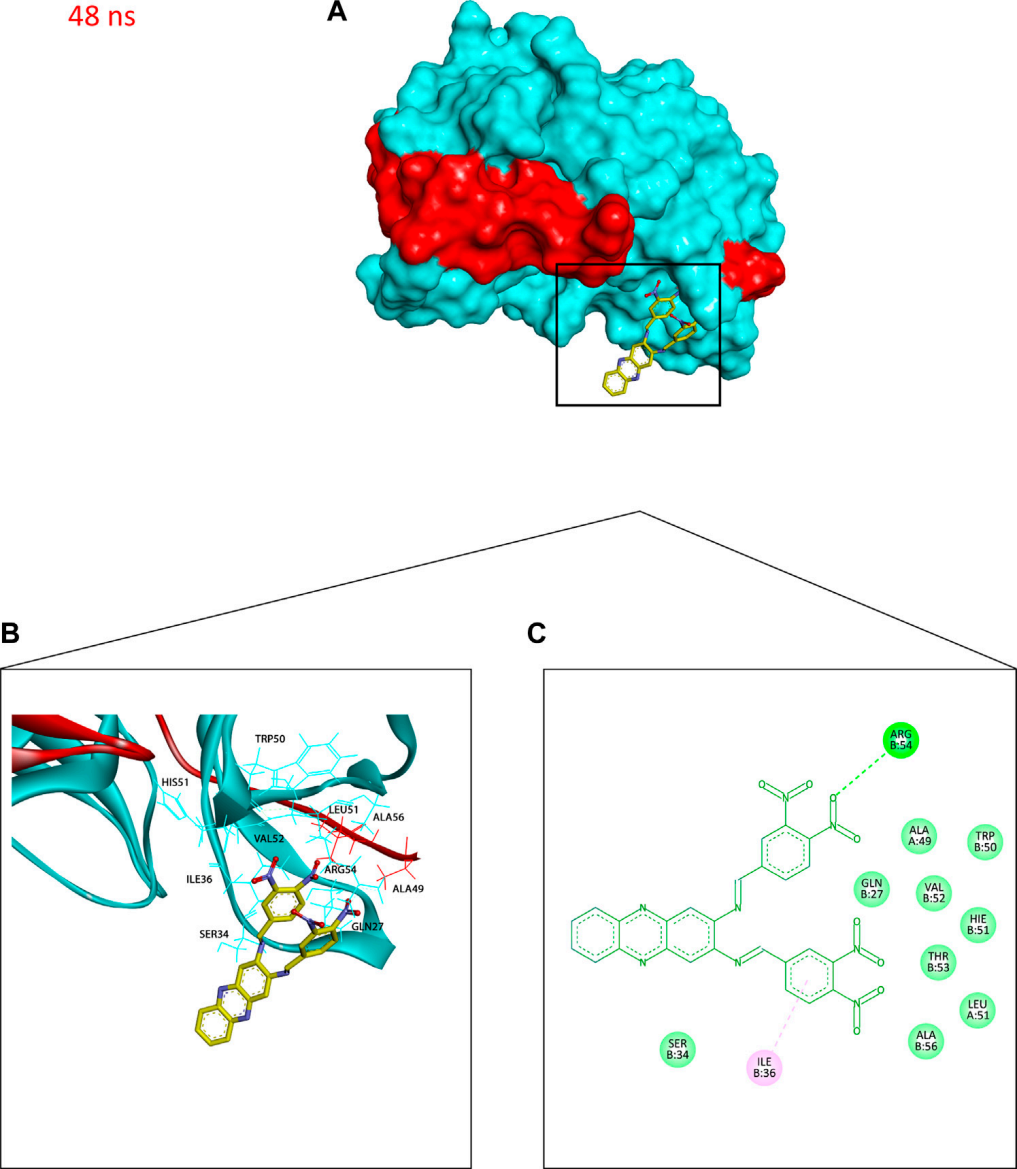
Binding conformation of compound **4** (stick representation with yellow carbons) at the catalytic binding site of DENV2 NS2B-NS3 protease (cyan for the NS3pro chain and red for the NS2B chain) at 48 ns showing the phenazine ring system protruding out of the protease binding pocket which caused the slight elevation in its RadGyr reading. **(A)** Surface presentation of the protease. **(B)** 3D representation of the interacting amino acid residues. **(C)** 2D representation of the interacting amino acid residues. The frame was extracted using UCSF Chimera 1.15 and represented using BIOVIA Discovery Studio.

Additionally, to assess the dynamic evolution and the stability of compounds **4** and **5** in complex with the NS2B-NS3 protease model of DENV2, molecular dynamics simulations were carried out for 50 ns. Generally, both systems demonstrated stability and consistent RMSD values with an average of 1.65 ± 0.17 and 1.71 ± 0.22 Å for **4**- and **5**-NS2B-NS3 protease, respectively, with a slight increment to a maximum of 3.06 Å in compound **5** complex at around 27.5 ns (Figure 8A). The slight elevations in the RMSD at 27.5 ns and the RMSF of GLU19 of compound **5** are attributed to its continuous conformation change between the S1 and S2 sub-pockets of the protease, yet it was not seen to leave the main binding pocket (Figure 9). Likewise, the RMSF profiles (Figure 8B) of NS2B-NS3 protease in complex with both compounds displayed comparable fluctuation patterns of the amino acid residues with higher fluctuation for compound **5** seen with GLU19 at around 3.7 Å. In addition, RadGyr analysis also showed a coherent rigidity and compactness of the systems (Figure 8C). The average RadGyr for compound **4**’s and **5**’s systems were 14.13 ± 0.20 and 14.11 ± 0.16 Å, respectively, and both systems were seen to fluctuate within less than 0.9 Å. Slight elevations were noticed between 48 and 49 ns for the system with compound **4** peaking at 14.40 Å, and for compound **5**’s system to around the same value between 49 and 50 ns. The slight elevation in the RadGyr profile in the case of compound **4** is due to the phenazine ring system protruding out of the protease binding pocket (Figure 10).

The H-bond interaction analyses of both systems at the catalytic binding site of the protease demonstrated that compound **4** was able to make up to five H-bonds throughout the 50 ns simulation time (Figure 8D). However, there was no persistent H-bond formed between any of the possible HA of compound **4** in contrast to that proposed by the docking. The two H-bond interactions with SER135 (one of the catalytic residues) and ARG54 with low occupancies of 0.21 and 0.10%, respectively demonstrated in Supplementary Table S3 are attributed to the oxygen and nitrogen atoms of compound **4** acting as acceptors. On the other hand, compound **5** exhibited a maximum of four H-bond interactions throughout the 50 ns simulation, with their frequency higher than compound **4** (Figure 8E). Six H-bond interactions at the binding site of the protease with higher occupancies were observed especially with HIS51 (11.57%), which is a catalytic residue of the protease in addition to VAL52 (1.83%), GLY55 (0.76%), GLY133 (0.61%, total), and ARG54 (0.11%). These six interactions are attributed to two oxygen atoms of compound **5** acting as acceptors, and some hydrogens atoms of the **5**’s oxygens and nitrogens acting as donors (Supplementary Table S3). The low occupancies of the H-bonds suggested that the H-bond interactions do not play an important role in the binding of the ligands. This is supported by the MM-PBSA energy components analyses that indicated the van der Waals interactions are the main contributors to the stability of the compounds in the protease binding site. MM-PBSA calculation is also agreeable to the other predictions that compound **4** has a better affinity towards the binding to the protease with ΔG_bind_ of -22.53 ± 4.21 kcal/mol than compound **5** (-17.01 ± 3.14 kcal/mol) (Supplementary Table S4).

## 4 Discussion

Dengue type-2 (DENV2) is the serotype most commonly found endemic in South East Asia (Halstead, 2006). Still, no specific drug clinically inhibits viral replication with a clear understanding of its molecular mechanism. This might be due to the limited availability of a 3D crystal structure of the virus that is required for further investigations. For example, the NS2B-NS3 protease of DENV2 is only available as an apo-enzyme (Erbel et al., 2006). Therefore, there is a limitation in confirming the coordinate of the ligand binding when the structure-based drug design is implemented. Lately, a crystal structure of DENV2 NS2B-NS3 protease complex with peptide inhibitor from mung bean was proven invalid. Hence, all related publications were retracted. Fortunately, homology modelling has been performed to solve this problem by utilising the DENV2 apo-enzyme structure with the ligand from West Nile Virus (WNV) NS2B-NS3 protease crystal structure (Wichapong et al., 2010). This model was successfully used in finding hits to lead in a few projects of DENV2 NS2B-NS3 protease inhibitor discovery bearing thioguanine (Abduraman et al., 2018; Hariono et al., 2019), benzofuranone (Sulaiman et al., 2019), and malabaricone-acylphenol (Sivasothy et al., 2021). Consequently, the homology model of DENV2 NS2B-NS3 protease structure was chosen in designing the corresponding target inhibitor from the nitro-benzylidene phenazine compound.

A structure-based design strategy was adopted based on the biological potential of compound **1** bearing *p*-hydroxyethyl benzoate ester linked to benzimidazole rings by hydrazine bridges, albeit at low micromolar potency. Analogue **22a** was obtained by modifying the nitrophenyl ring and was shown to be active against the protease enzyme. Subsequently, screening of the National Cancer Institute (NCI) compounds database identified a series of *N*-heterocyclic compounds linked to nitrophenyl rings with inhibitory activity against DENV2 NS2B-NS3 protease. These compounds are associated with pharmacophore features like hydrazine bridges (HBD/HBA), nitrophenyl (HBA/H), and *N*-heterocycle (HBA/H), up to the compound having nitro-benzylidene phenazine structure.

Compound **22a** was used as a reference compound to validate the generated pharmacophore model, which corroborated the latest findings upon retrospective validation. The developed pharmacophore model allowed the identification of a true positive DENV2 NS2B-NS3 protease inhibitor than its false positive compound. A validated pharmacophore model was then used to screen the hits of DENV2 NS2B-NS3 protease inhibitor from a series of nitro-benzylidene phenazine analogues. The nitro-benzylidene phenazine was designed based on the consensus between the common pharmacophore features of DENV2 NS2B-NS3 protease inhibitor and its synthesis feasibility, resulting in 13 designed ligands. Despite the identification of only two ligands (**4** and **5**) as hits from the structure-based pharmacophore screening, they are predicted to become promising candidates for further optimisation.

Results from molecular docking revealed low values in their ΔG_bind_ for both compounds **4** and **5** at the DENV2 NS2B-NS3 protease catalytic binding site. This suggested the high affinity of the hits towards the enzyme. Furthermore, the binding mode of the two hits demonstrated similar key interactions as in the case of **22a** with the amino acid residues lining the binding pocket. Designing new ligands that could occupy the catalytic region might interfere with substrate binding ability and thus could be a potential target site to design such specific ligands that can interfere with the protein function.

In general, ligands can exhibit false positive activities in an *in vitro* assay. Herein, prediction using the PAINS filter was applied. Undetected PAINS with false-positive activities are often due to covalent modifications, redox effects, chelation, autofluorescence, or degradation. The study of pharmacokinetics refers to the investigations of drug-likeness, mutagenic/toxicity and pharmacokinetic predictions on the potential new hits before entering the next process; lead optimisation.

One of the main factors that limit oral drug absorption might be the large structure of compounds **4** and **5**. Furthermore, they show possible human intoxication and mutagenic effects. This could be due to the presence of a nitro aromatic group which had been identified to possess a mutagenic effect (Chin Chung et al., 2011). The nitro group is a unique functional group in medicinal chemistry as a strong electron-withdrawing and a relatively polar group. Although found in medicines for a long time, the nitro group has toxicity issues due to metabolic liabilities. Therefore, it is often considered a structural alert or toxicophore, leading to mutagenicity and genotoxicity.

However, numerous nitro-drugs take advantage of bio-reductive activation mechanisms, mainly in the antibacterial and antiparasitic fields. Recently, renewed interest in nitro drugs has led to the approval of fexinidazole for the treatment of human African trypanosomiasis (HAT), a neglected tropical disease. Due to its unique properties, the nitro group has been found to have great interest in the development of anticancer, antitubercular and antiparasitic agents, as well as hypoxia-activated prodrugs. In addition, nitro groups have been found in the development of self-immolative spacers (linkers) to overcome limitations for drug delivery and have attracted wide interest in medicinal chemistry. In this context, many studies have been undertaken on the structure-mutagenicity or structure-genotoxicity relationships of drugs containing nitro groups (https://www.mdpi.com/journal/pharmaceuticals/special_issues/Nitro_Drugs).

**FIGURE 11:**
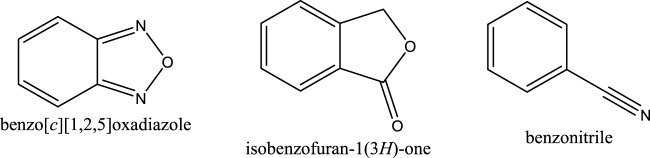
The structures of benzo [c][1,2,5]oxadiazole, isobenzofuran-1(3H)-one, and benzonitrile.

During the distribution, both hits (compounds **4** and **5**) were found unable to reach the maximum dose due to their low VDss. It was observed that both hits are capable of inhibiting or inducing the CYP enzyme activities in combination medicines that include other active ingredients in the presence of the compound with no significant changes in the excretion process. As shown in Supplementary Table S5, the % efficiency of both hits in terms of drug-like structure, toxicity, absorption, distribution, metabolism and excretion revealed less than 100%, with only 52–55% efficiency for nitro-benzylidene phenazine to be considered for further lead optimisation. This result suggested substituting some functional groups with their bioisostere in future work. For example, the nitro group has bioisosteres such as trifluoromethyl (CF_3_) group in CB_1_ receptor positive allosteric modulation (Tseng et al., 2019). An earlier study has identified the bioisostere of a nitro-aromatic group with some groups including benzo [c][1,2,5]oxadiazole, isobenzofuran-1(3*H*)-one, and benzonitrile (Meanwell, 2011) (Figure 11).

As compounds **4** and **5** are interesting and suggested for further lead optimisation, the binding stability of those compounds in the DENV2 NS2B-NS3 protease active site should be investigated. Molecular dynamics simulations demonstrated that compounds **4** and **5** exhibited excellent stability when in complex with the DENV2 NS2B-NS3 protease model. Due to the continuous conformation change of compound **5** between the S1 and S2 sub-pockets of the protease that was shown earlier, compound **5** was seen to contribute through higher H-bond interaction occupancies, especially with HIS51, which constitutes one of the DENV protease catalytic residues. Moreover, although compound **4** showed an H-bond interaction with SER135 with limited occupancy, it can be concluded that both compounds have their total free energy of binding contributions through vdW and non-polar interactions with the amino acids lining the main binding pocket of the protease. All these findings confirm and support the results obtained from the molecular docking earlier, where ARG54 does not contribute mainly through H-bonds with both ligands rather than through non-bonding interactions. Moreover, the unfavourable acceptor-acceptor interaction between PRO132 and compound **5** that was seen from the docking results might have influenced its higher ΔG_bind_ from the MM-PBSA analysis, not to mention that both the molecular docking and dynamics studies agree that compound **4** seems to have a higher binding affinity towards the protease than compound **5**.

## 5 Conclusions

A pharmacophore model for DENV2 NS2B-NS3 protease inhibitor has been generated from the docking pose of ethyl 4-hydroxy-3,5-*bis*((2-(4-nitrophenyl)hydrazinylidene)-methyl)benzoate (**22a**) in the binding pocket of DENV2 NS2B-NS3 protease. The pharmacophore model employing 5 HBAs, 3 HBDs and 1 H was able to identify two hits from the nitro-benzylidene phenazine series. These two hits show potential binding in the DENV2 NS2B-NS3 protease based on their molecular docking results. However, after drug-likeness, mutagenic/toxicity and pharmacokinetic prediction, these two compounds resulted in 52–55% efficiency as drug candidates while exhibiting stability at the catalytic pocket of the protease mainly through vdW and non-polar interactions. The challenge of these hits is due to their mutagenic-toxic effect on the nitro aromatic group. Therefore, further studies should consider the replacement of this group with its bioisostere to improve the bioactivity against DENV2 NS2B-NS3 protease.

## Data Availability

The original contributions presented in the study are included in the article/Supplementary Material, further inquiries can be directed to the corresponding authors.
