# Supplementary material for: Computational study of nitro-benzylidene phenazine as dengue virus-2 NS2B-NS3 protease inhibitor
Source: Front Mol Biosci. 2022 Nov 17;9:875424. doi: 10.3389/fmolb.2022.875424 (PMC9715268; doi:10.3389/fmolb.2022.875424)
Supplement: Supplementary file 1 [file DataSheet1.PDF]

Table S1.

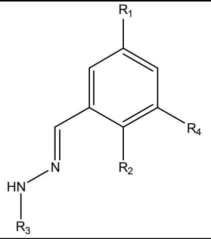

Entry	Cmpd	R1	R2	R3	R4	IC_{50} (μM) Inhibitory rate at 100 μM
1	1	CO_2Et	OH	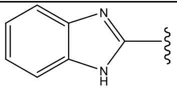	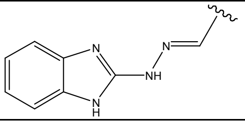	13.12±1.03
2	22m	CO_2Et	H	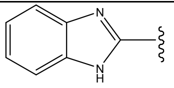	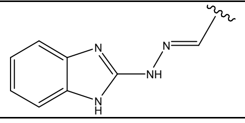	29.53±2.15

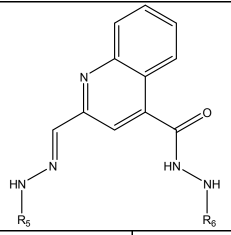

Entry	Cmpd	R5	R6	IC_{50} (μM) Inhibitory rate at 100 μM
3	23a	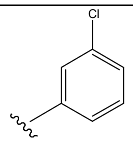	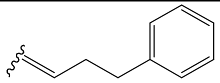	29.04±1.78
4	23b	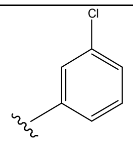	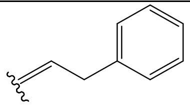	28.12±1.96
5	23d	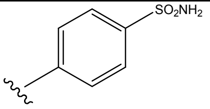	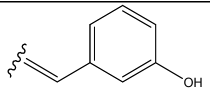	7.84±0.94
6	23e	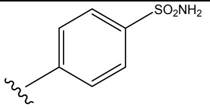	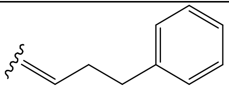	36.02±3.05
7	23g	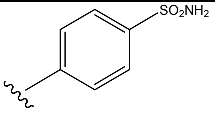	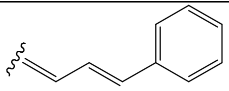	7.46±1.15
8	23h	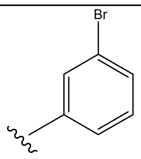	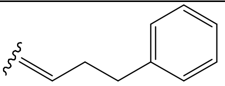	`19.93±0.98

9	23i	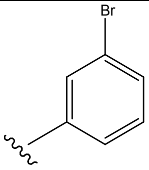	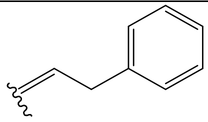	9.45±0.78
10	23j	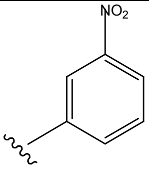	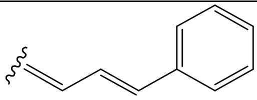	21.96±2.05
11	23l	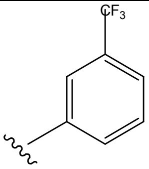	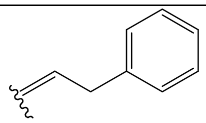	19.8±1.15
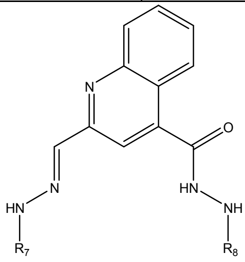				
Entry	Cmpd	R7	R8	IC_{50} (μM) Inhibitory rate at 100 μM
12	24g	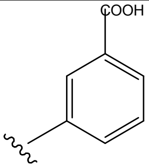	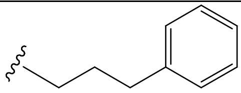	41.24±5.53
12	24h	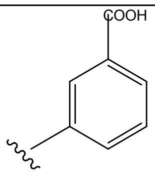	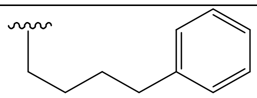	35.28±4.36

Table S2

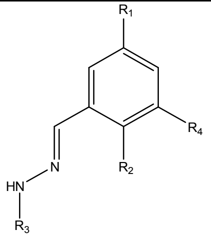

Entry	Cmpd	R1	R2	R3	R4	IC_{50} (μM) Inhibitory rate at 100 μM
1	22b	CO-$_2$Et	OH	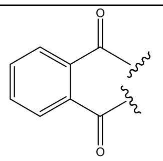	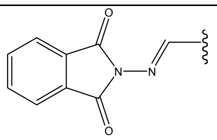	> 100
2	22m	CO-$_2$Et	OH	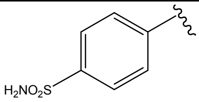	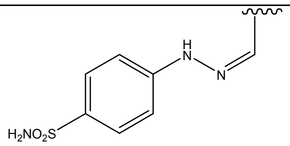	NA

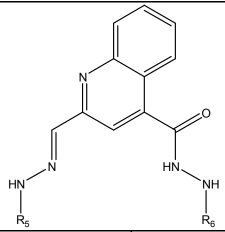

Entry	Cmpd	R5	R6	IC_{50} (μM) Inhibitory rate at 100 μM
3	23f	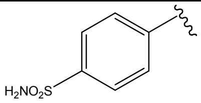	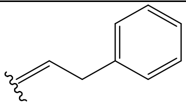	> 100
4	23k	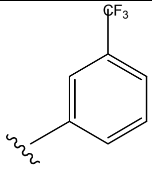	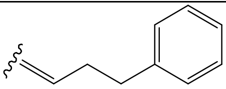	>100
5	23m	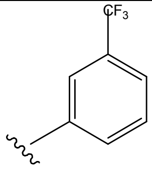	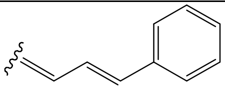	>100

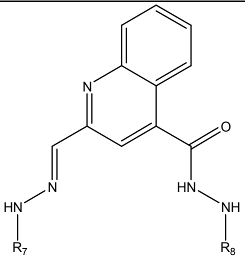

Entry	Cmpd	R7	R8	IC$_{50}$ (μM) Inhibitory rate at 100 μM
6	24g	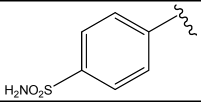	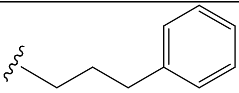	>100
7	24b	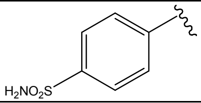	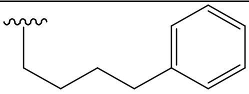	>100
8	24c	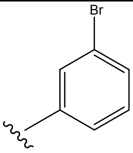	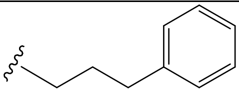	>100
9	24d	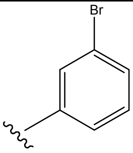	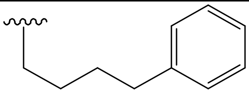	>100
10	24e	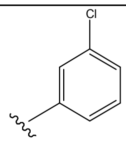	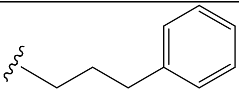	>100
11	24f	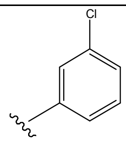	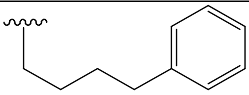	NA
