# Supplementary material for: Computational study of nitro-benzylidene phenazine as dengue virus-2 NS2B-NS3 protease inhibitor
Source: Front Mol Biosci. 2022 Nov 17;9:875424. doi: 10.3389/fmolb.2022.875424 (PMC9715268; doi:10.3389/fmolb.2022.875424)
Supplement: Supplementary file 2 [file DataSheet2.docx]

| **Table S3.** Hydrogen bond analyses of the simulated systems; compound **4**-NS2b/NS3pro and compound **5**-NS2b/NS3pro. | | | | | | |  |  |
| --- | --- | --- | --- | --- | --- | --- | --- | --- |
| **Complex** | | **Acceptor** | **DonorH** | **Donor** | **Occupancy (%)** | **Average Distance (Å)** | **Average Angle (Å)** | |
| C4-NS2b-NS3pro | | C4@N2 | SER135@HG | SER135@OG | 0.21 | 2.86 | 154.55 | |
|  | | C4@O6 | ARG54@HH11 | ARG54@NH1 | 0.1 | 2.90 | 148.88 | |
| C5-NS2b-NS3pro | | HIS51@O | C5@H2 | C5@O6 | 11.57 | 2.67 | 163.47 | |
|  | | VAL52@O | C5@H2 | C5@O6 | 1.83 | 2.69 | 163.83 | |
|  | | GLY55@O | C5@H2 | C5@O6 | 0.76 | 2.72 | 153.51 | |
|  | | C5@O2 | GLY133@H | GLY133@N | 0.31 | 2.90 | 147.32 | |
|  | | C5@O3 | GLY133@H | GLY133@N | 0.30 | 2.91 | 144.59 | |
|  | | ARG54@O | C5@H2 | C5@O6 | 0.11 | 2.73 | 156.91 | |

| **Table S4.** Predicted energy components and binding affinities of compounds **4** and **5** compounds towards DENV2 NS2B-NS3 protease using MM-PBSA approach for the last 5 ns of the molecular dynamics simulations. | | |
| --- | --- | --- |
| **MM-PBSA Energy Component** | **Energy Value (kcal/mol) ± SD per Complex** | |
|  | **C4-NS2B-NS3pro** | **C5-NS2B-NS3pro** |
| van der Waals Energy (ΔE_vdW_) | -34.54±5.52 | -25.19±5.00 |
| Electrostatic Energy (ΔE_EEL_) | -7.45±8.65 | -2.04±2.74 |
| Polar Solvation Energy (ΔG_PB_) | 22.58±10.41 | 12.56±4.23 |
| Non-Polar Solvation Energy (ΔE_npolar_) | -3.11±0.43 | -2.34±0.45 |
| Total Gas Phase Free Energy (ΔG_gas_) | -41.99±13.09 | -27.23±6.16 |
| Total Solvation Phase Free Energy (ΔG_solv_) | 19.47±10.02 | 10.22±3.86 |
| Total Binding Free Energy (ΔG_bind_) | -22.53±4.21 | -17.01±3.14 |

| **Table S5.** The efficiency percentage of the hits in drug-like structure, toxicity, absorption, distribution, metabolism and excretion. | | | | | | | | | | | | | | |
| --- | --- | --- | --- | --- | --- | --- | --- | --- | --- | --- | --- | --- | --- | --- |
| **Ligand** | | **Drug-like structure** | **Toxicity** | | **Absorption** | | **Distribution** | | **Metabolism** | | **Excretion** | | **Mean** | |
| 4 | 50 | | | 40 | | 57 | | 25 | | 57 | | 100 | | 55 |
| 5 | 50 | | | 40 | | 43 | | 50 | | 29 | | 100 | | 52 |
